# Simultaneous Ablation of Uterine Natural Killer Cells and Uterine Mast Cells in Mice Leads to Poor Vascularization and Abnormal Doppler Measurements That Compromise Fetal Well-being

**DOI:** 10.3389/fimmu.2017.01913

**Published:** 2018-01-08

**Authors:** Nicole Meyer, Thomas Schüler, Ana Claudia Zenclussen

**Affiliations:** ^1^Experimental Obstetrics and Gynecology, Medical Faculty, Otto-von-Guericke University, Magdeburg, Germany; ^2^Institute of Molecular and Clinical Immunology, Medical Faculty, Otto-von-Guericke University, Magdeburg, Germany

**Keywords:** angiogenesis, fetal development, intrauterine growth restriction, placenta, mast cells, natural killer cells, ultrasound imaging, Doppler measurements

## Abstract

Intrauterine growth restriction (IUGR) is a serious pregnancy complication with short- and long-term health consequences. The mechanisms underlying this condition are not well understood. Animal models are the basis for understanding the causes of IUGR and for developing useful therapeutic strategies. Here, we aimed to ascertain the *in utero* growth of fetuses from NK (natural killer cells)/MC (mast cells)-deficient mothers that give birth to growth-restricted pups and to determine the time point at which IUGR starts. We used high frequency ultrasound imaging to follow-up fetal and placenta size and employed Doppler measurements to document blood supply to the fetus in females that were deficient for NK cells and MCs. In mice lacking NKs and MCs, we observed significantly reduced implantation sizes from mid gestation onward, which was further associated with smaller placentas. Additionally, NK/MC-deficiency was associated with absent and reversed end diastolic flow in umbilical arteries of the fetuses and an increased systolic/diastolic ratio as well as an elevated resistance index. Together, our results indicate that NKs/MCs promote blood flow, placental growth, and subsequent fetal development. The results of this study offer new insights as to how fetal growth is affected *in vivo* in NK/MC-deficient mice, whose pups are growth restricted at birth. The use of IUGR models and modern technologies enabling the *in vivo* follow-up of fetal development are important tools for understanding mechanisms behind pregnancy complications that in the future may lead to the development of effective therapies.

## Introduction

Intrauterine growth restriction (IUGR) is defined by the inability of the fetus to reach its genetically determined growth potential due to a pathological growth restriction *in utero*. A common definition is a weight below the 10th percentile (1–4), indicating that the affected fetus has a lighter weight than 90% of other fetuses at the same gestational age. A special degree of severity exists if the fetal weight is below the 5th percentile (5). Reasons for the development of IUGR can arise from the maternal or the fetal site, including genetic failure, infections, maternal smoking, and often a defect in proper placental development (6). During normal pregnancy uterine vascular changes, including angiogenesis and remodeling of the spiral arteries (SAs), are crucial to ensure the continuous blood flow to the placenta which has to adapt in order to meet the requirements of the growing nutrients and oxygen (7). Inadequate vascular changes and impaired SA remodeling in human can cause preeclampsia, IUGR, preterm birth, or miscarriage (8–10). The mechanisms and mediators affecting fetal development have not yet been conclusively determined. The failure of a fetus to achieve its full growth potential is accompanied by increased rates of perinatal mortality as well as short- and long-term morbidity (3). It is associated with the development of serious metabolic and cardiovascular diseases like diabetes, obesity, osteoporosis, hypertension, heart diseases, and stroke (11–13). Increased mortality and morbidity of growth-restricted newborns not only leads to physical and mental stress for the patients but also associated with enormous costs for our health system. Therefore, it is of vital importance to understand the mechanisms behind IUGR in order to elaborate rational approaches to prevent it and foster normal pregnancy.

It is long known that cells of the innate immune system prepare the uterus for pregnancy (14). In particular, uterine (u)NKs and uMCs promote vascular remodeling by secreting factors that foster apoptosis of uterine smooth muscle cells thereby contributing to their replacement replaced by trophoblasts. We have recently reported that uNKs and uMCs seem to have contributory functions and counteract each other in order to ensure that such a relevant process does take place (15). We further established a mouse model lacking uNKs and uMCs to investigate their impact on pregnancy outcome. Female uNKs/uMCs-deficient mice exhibited strongly impaired SA remodeling as compared to mice lacking either cell type. Furthermore, more than half of the pups were growth restricted at birth. Whether the observed phenotype is a direct consequence of impaired blood flow and reduced levels of maternal blood reaching the placental labyrinth is not known. Here, we used the high frequency ultrasound Vevo^®^ 2100 system as a non-invasive tool to quantify implantation and placental dimensions at different pregnancy time points and to evaluate the blood flow in maternal and fetal arteries in these mice. By using this technology, we can also follow-up single implantations in real time and determine at exactly which time point the fetuses start to suffer from growth restriction and whether this may be consequence of a slower blood flow or more resistant arteries.

We found that uNK/uMC-deficiency impacted pregnancy from mid gestation onward; at this time point, SA remodeling should be completed. We observed smaller implantation sizes and reduced placental dimensions. Additionally, we could detect absent or reversed end diastolic flow in the *Arteria umbilicalis* (UmAs) of some fetuses of uNK/uMC-deficient mice that lead to an increased systolic/diastolic ratio and an increased resistance index of the arteries compared to WT mice. These are signs of poor vascularization that compromise fetal well-being and imposingly confirm that these two cell types are pivotal components of uterine angiogenesis.

## Materials and Methods

### Mouse Model

This study was carried out in accordance with the recommendations of the Ministery of Saxony-Anhalt, Germany. The protocol was approved by the “Landesverwaltungsamt Sachsen Anhalt: 42502-2-1296UniMD.” Mice were housed in our barrier facility with a 12-h light/dark cycle and received food and water *ad libitum*. MC-deficient C57BL/6J-Cpa3^Cre/+^ (Cpa3^Cre/+^) mice and their WT controls C57BL/6J-Cpa3^+/+^ (Cpa3^+/+^) were kindly provided by HR Rodewald (Heidelberg, Germany) (16) and bred in our facilities. 6- to 8-week-old females were allogeneically mated with BALB/c males (Charles River). The day of vaginal plug detection was defined as gestation day (gd) 0. Cpa3^Cre/+^ females receive 0.25 mg (250 µl) anti-CD122 (BioLegend, London, United Kingdom) *via* intraperitoneal injection immediately after the detection of the copulation plug, to deplete peripheral and uNKs as described previously (17). Depletion of peripheral NKs with anti-CD122 was confirmed until gd18 (18 days after application) by analysis of different organs by flow cytometry. Additionally, uNKs absence was confirmed at gd10 (10 days after application) *via Dolichos biflorus* agglutinin staining (17). Control Cpa3^+/+^ females received 250 µl PBS. Females underwent ultrasound imaging at gd5, 8, 10, 12, and 14 and were sacrificed at gd14.

### Ultrasound Imaging

For ultrasound measurements the Vevo^®^ 2100 system (Visualsonics, Amsterdam, Netherlands), a non-invasion high-resolution imaging tool, was used. Mice were anesthetized with isoflurane (Baxter, Burgdorf, Germany) in the knock down box and afterwards taped with surgical tape (3M, Neuss, Germany) with the paws on electrode gel (Parker Laboratories, NJ, USA)-coated copper areas of a heating platform. Thereby the mouse was positioned with the face toward the scientist. ECG, body temperature, and respiratory physiology were controlled at all times. To prevent dry eyes, eye creme (Bayer, Leverkusen, Germany) was used. The hair on the abdomen was removed by employing depilatory cream (Reckitt Benckiser, Hull, United Kingdom). Then pre-warmed ultrasound gel (Gello GmbH Geltechnik, Aheus, Germany) was applied on the depilated skin. First, the bladder was identified with the transducer (MS550D-0421) and used as reference point and then the transducer was moved to the left and the right site of the abdomen to trace implantations. B-Mode was used to visualize anatomical structures in 2D grayscale image. Color Doppler Mode was used to visualize blood flow in the *Arteria uterina* (UA) and *Arteria umbilicalis* (UmA). Pulse-wave (PW) Doppler Mode was used to quantify the blood flows through the vessels. Angle between the direction of the blood flow and transducer was set at 70° for the UAs and at 45° for the UmAs. Peak systolic velocities (PSVs) and end diastolic velocities (EDVs) of UAs and UmAs were recorded (average of 10 PSV and associated EDV measurements per mother/fetus was used) and analyzed with the Visualsonics software. The software automatically calculated the resistance Index [RI; RI = (PSV − EDV)/PSV] and the pulsatility index [PI; PI = (PSV − EDV)/velocity time integral]. Ultrasound examinations were performed at gd5, 8, 10, 12, and 14 and all implantations/litters found within the mothers were imaged. Mice were never exposed longer than 1 h to gaseous anesthesia.

### Weight Determination

Fetal and placental weights were measured with a micro scale (Kern & Sohn GmbH, Balingen, Germany) at gd14. Feto-placental indexes (FPIs) were calculated by dividing the weight of the fetus by the placental weight.

### Statistical Analysis

The D’Agostino Pearson-Omnibus normality test was used to assess normality of the data sets. Data are presented as means ± SEM. The number of mice, samples, the statistical test and the *P* values are indicated in each figure legend. Statistical analyses were performed with GraphPad Prism 5.0.

## Results

### Mice Devoid of NK Cells and MCs Present Smaller Implantation Sizes Beginning at Mid Gestation As Analyzed by High Frequency Ultrasound

**Figure 1:**
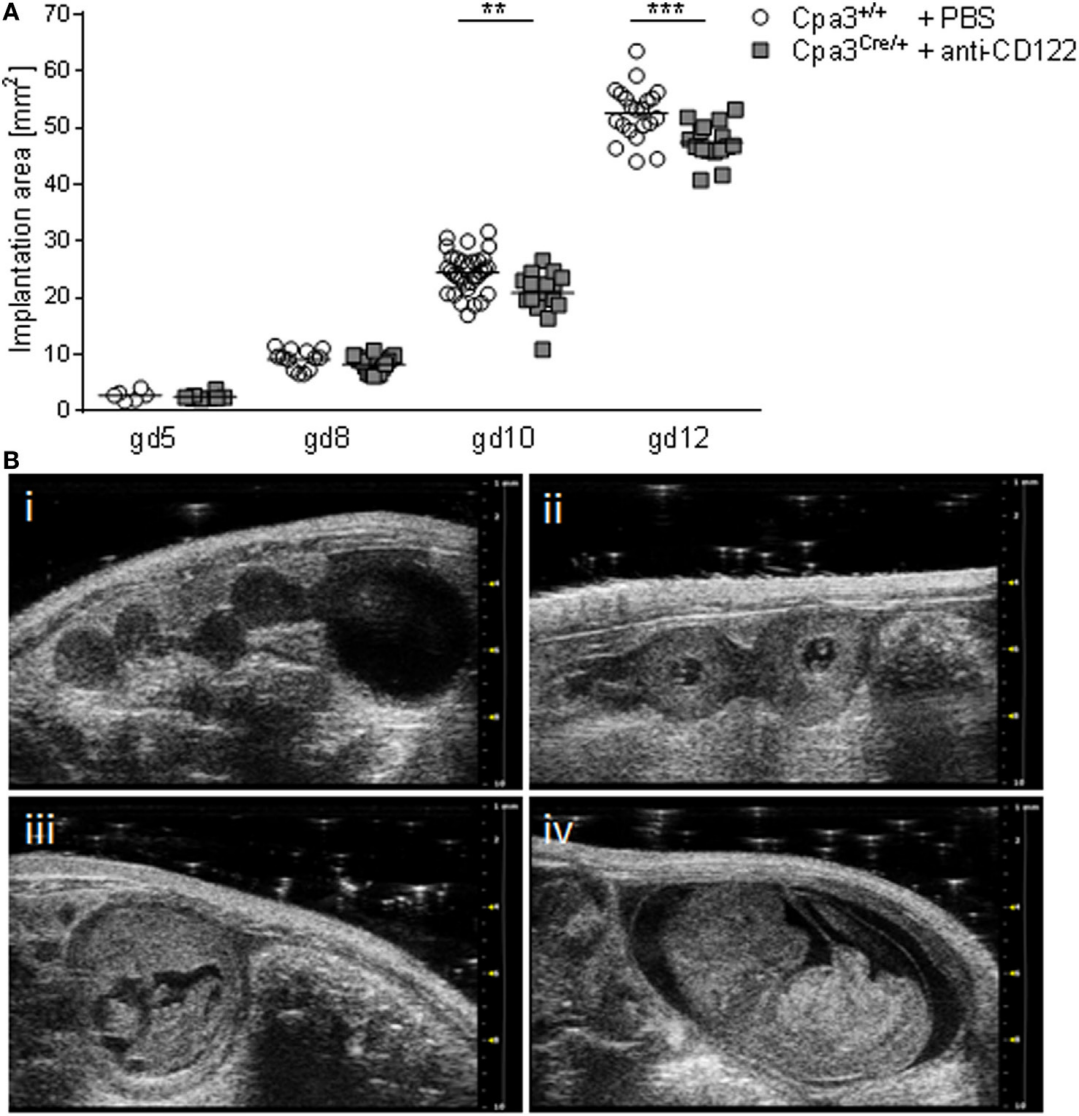
Comparison of implantation areas at gd5, 8, 10, and 12. **(A)** Implantation areas from WT Cpa3^+/+^ + PBS mice (mice *n* = 2–5, implantations *n* = 6–31 per day) and MC/NK-deficient Cpa3^Cre/+^ + anti-CD122 mice (mice *n* = 3, implantations *n* = 8–16 per day) at gd5, 8, 10, and 12. Results are presented as individual values for each single implantation and mean. Statistical differences were obtained using unpaired *t*-test (**P < 0.01, ***P < 0.001). **(B)** Representative ultrasound images from Cpa3^+/+^ + PBS mice at gd5 (i), gd8 (ii), gd10 (iii), and gd12 (iv). gd, gestation day; WT, wild type; MC, mast cell; NK, natural killer cell.

Given the fact that the progeny of NK/MC-deficient mothers suffers from IUGR (17), we aimed to determine the earliest time point of IUGR manifestation *in utero*. For this, we documented implantations at days 5, 8, 10, and 12 of anti-CD122-treated Cpa3^Cre/+^ (NK/MC-deficient) and PBS-treated control Cpa3^+/+^ (WT) mice by employing high frequency ultrasound with the Vevo^®^ 2100 system. After day 12, implantations were too large to be measured correctly. Measurements obtained in the B-Mode show a similar implantation area size among the groups at gd5 and 8, whereas a statistically significant smaller implantation area could be observed in NK/MC-deficient mice compared to controls at gd10 (*P* < 0.01). On average, the implantation size of NK/MC-deficient mice at gd10 was 15% smaller compared to controls and 10% at gd12, the differences were statistically significant (*P* < 0.001, Figure 1A), and this correspond to the already reported differences in weight of fetuses and placentas (15). Representative ultrasound images show implantations at gd5 (Figure 1B, i), gd8 (Figure 1B, ii), gd10 (Figure 1B, iii), and gd12 (Figure 1B, iv) of WT mice. Our results indicate that growth restriction starts during mid gestation.

### NK/MC-Deficient Mice Present Reduced Placental Dimensions at gd10 and 12

**Figure 2:**
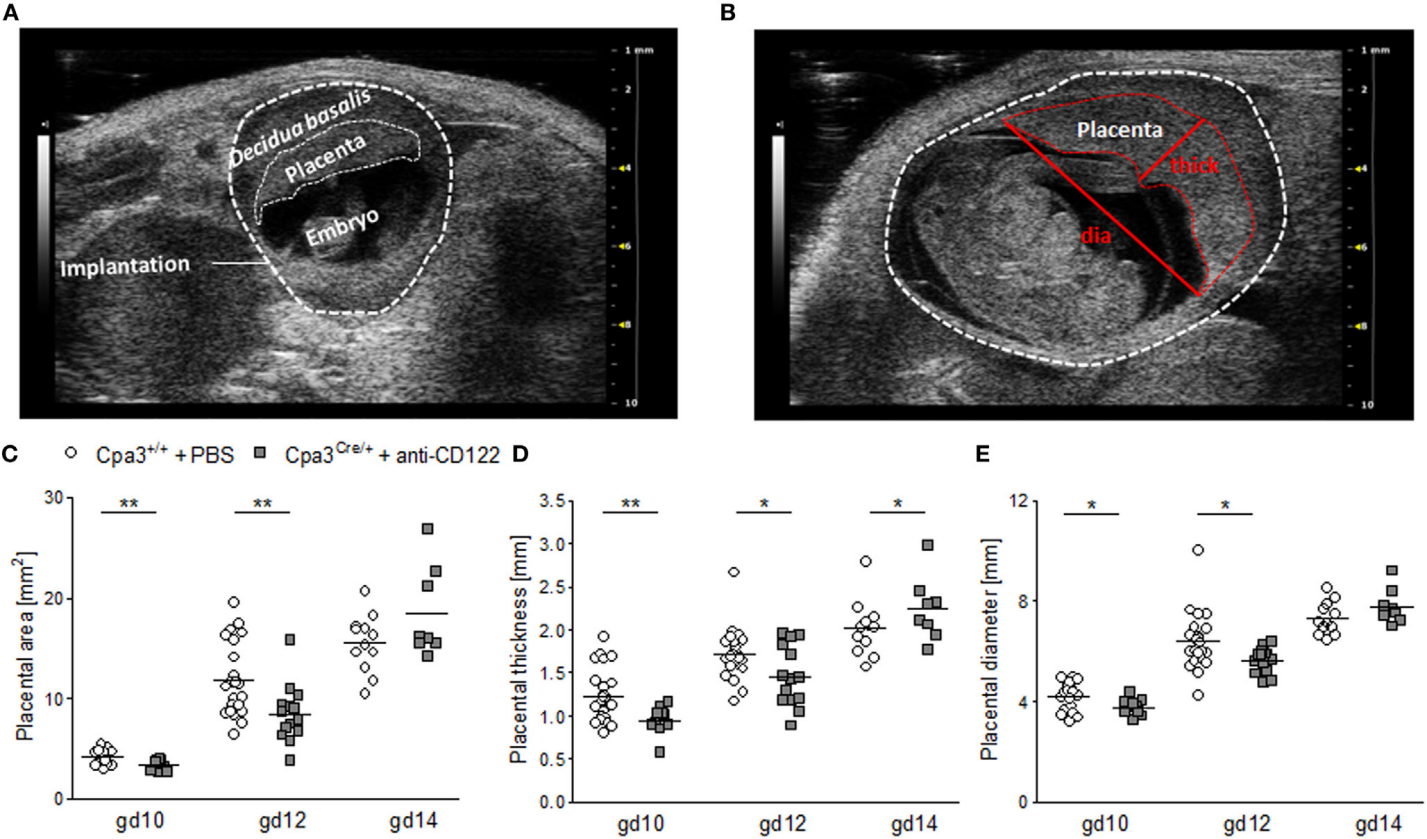
Placental measurements at gd10, 12, and 14. **(A)** Representative ultrasound image of a WT implantation at gd10 showing the *decidua basalis*, the placenta, and the embryo. **(B)** Representative ultrasound image of a WT implantation at gd12 showing placental thickness (thick) and placental diameter (dia). Placental area **(C)**, placental thickness **(D)**, and placental diameter **(E)** from WT Cpa3^+/+^ + PBS mice (mice *n* = 3–5, placentas *n* = 12–22 per day) and MC/NK-deficient Cpa3^Cre/+^ + anti-CD122 mice (mice *n* = 3–4, placentas *n* = 8–14 per day) at gd10, 12, and 14. Results are presented as individual values for each single placenta and mean. Statistical differences were obtained using unpaired *t*-test (*P < 0.05, ** *P* < 0.01). gd, gestation day; WT, wild type; thick, thickness; dia, diameter; MC, mast cell; NK, natural killer cell.

To investigate whether smaller implantation areas of NK/MC-deficient mice were associated with differences in placenta size between the groups, placental measurements were performed at gd10 (Figure 2A), gd12 (Figure 2B), and gd14. Ultrasound placenta measurements are not possible before gd8 or after gd14, because of a too small or too large size, respectively. Placental area, placental thickness and placental diameter were determined (Figure 2B). Consistent with the smaller implantation size at gd10 and 12, placental areas (*P* < 0.01) (Figure 2C), placental thicknesses (*P* < 0.01; *P* < 0.05) (Figure 2D), and placental diameters (*P* < 0.05) (Figure 2E) were significantly reduced at gd10 and 12 in NK/MC-deficient mice. At gd14 placenta thicknesses were statistically increased (*P* < 0.05) in NK/MC-deficient mice. In the contrary, placental areas and placental diameters were comparable in both groups. Results indicate that smaller implantation sizes at gd10 and 12 are associated with smaller placenta dimensions; however, placental growth recovers until gd14.

### Fetuses from NK/MC-Deficient Mice Are Growth Restricted

**Figure 3:**
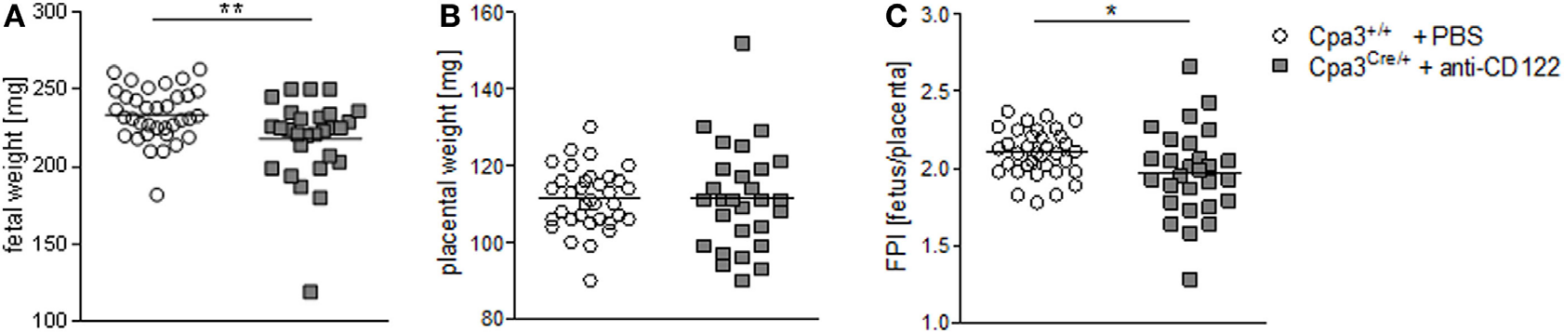
Fetal and placental weight measurements and feto-placental index (FPI) at gd14. Fetal weights **(A)**, placental weights **(B)**, and FPIs **(C)** from progeny of WT Cpa3^+/+^ + PBS mice (mice *n* = 4, fetus/placentas *n* = 35) and MC/NK-deficient Cpa3^Cre/+^ + anti-CD122 mice (mice *n* = 3, fetus/placentas *n* = 28) at gd14. Results are presented as individual values and mean. Statistical differences were obtained using unpaired *t*-test (*P < 0.05, ** *P* < 0.01). gd, gestation day; WT, wild type; MC, mast cell; NK, natural killer cell.

We found statistically significant smaller implantation sizes and placental areas at gd10 and 12 in NK/MC-deficient mice compared to WT mothers. Next, we asked whether reduced placental dimensions may correlated with fetal development. For this purpose, we recorded fetal weight at gd14. Additionally, we determined the placental weight to calculate the FPI by dividing the fetal weight through its placenta weight, which is an indirect measurement of placental function insufficiency (18). There was a statistically significantly lower (*P* = 0.01) fetal weight in NK/MC-deficient mice (218.0 ± 26.75) compared to WT mice (233.6 ± 17.02) (Figure 3A) and a comparable placental weight (Figure 3B) at gd14. Thus, FPI was statistically significantly lower (*P* = 0.05) in NK/MC-deficient mice (2.102 ± 0.149) compared to WT mice (1.972 ± 0.283) (Figure 3C). These results indicate that a reduced placental size during mid gestation in NK/MC-deficient mice is associated with fetal growth restrictions and placental insufficiency at gd14.

### NK/MC-Deficient Pregnant Mice Show Normal Uterine Artery Velocities As Measured by Doppler

**Figure 4:**
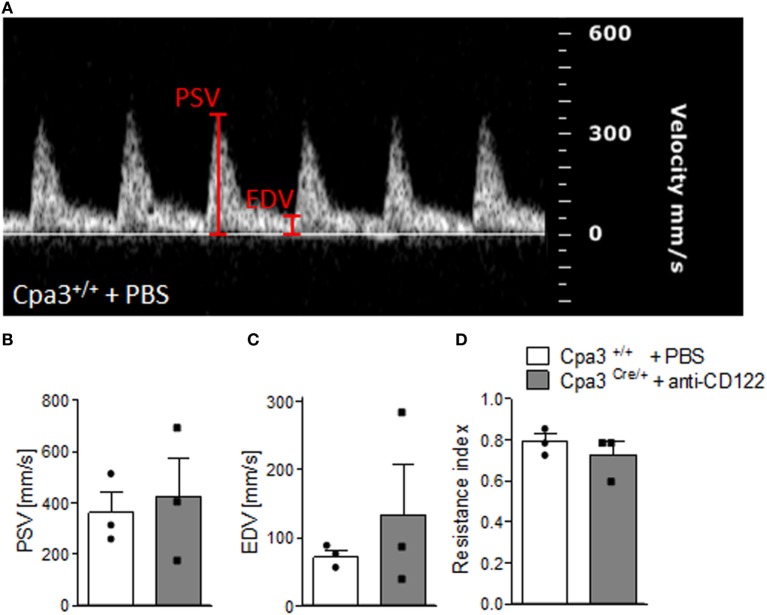
Analysis of uterine artery velocities at gd10. **(A)** Representative Pulse-wave Doppler images from WT Cpa3^+/+^ + PBS mice showing PSV and EDV. PSV **(B)**, EDV **(C)**, and resistance index **(D)** of uterine arteries from Cpa3^+/+^ + PBS (*n* = 3) and Cpa3^Cre/+^ + anti-CD122 (*n* = 3) mice at gd10 of pregnancy. Data are presented as mean with SEM. Statistical analysis was performed using the Mann–Whitney *U* test. gd, gestation day; WT, wild type; MC, mast cell; NK, natural killer cell; PSV, peak systolic velocity; EDV, end diastolic velocity.

We have recently reported that NK/MC-deficient female mice showed insufficiently remodeled SA in contrast to WT mice at gd10 and that this correlates with IUGR (17). As SA remodeling effects uteroplacental blood flow (19), we used high frequency ultrasound UA parameters to clarify whether insufficient SA remodeling and IUGR are reflected by abnormal blood flow from the mother to the fetus. PW Doppler images of NK/MC-deficient vs wild-type mice (Figure 4A) were used to calculate PSV (Figure 4B), EDV (Figure 4C), and the resistance index (Figure 4D). All measured parameters were comparable between the groups, indicating that insufficient SA remodeling and IUGR can occur without previous abnormalities in UA parameters as measured by ultrasound.

### Fetuses from NK/MC-Deficient Mothers Have Abnormal Umbilical Artery Velocities

**Figure 5:**
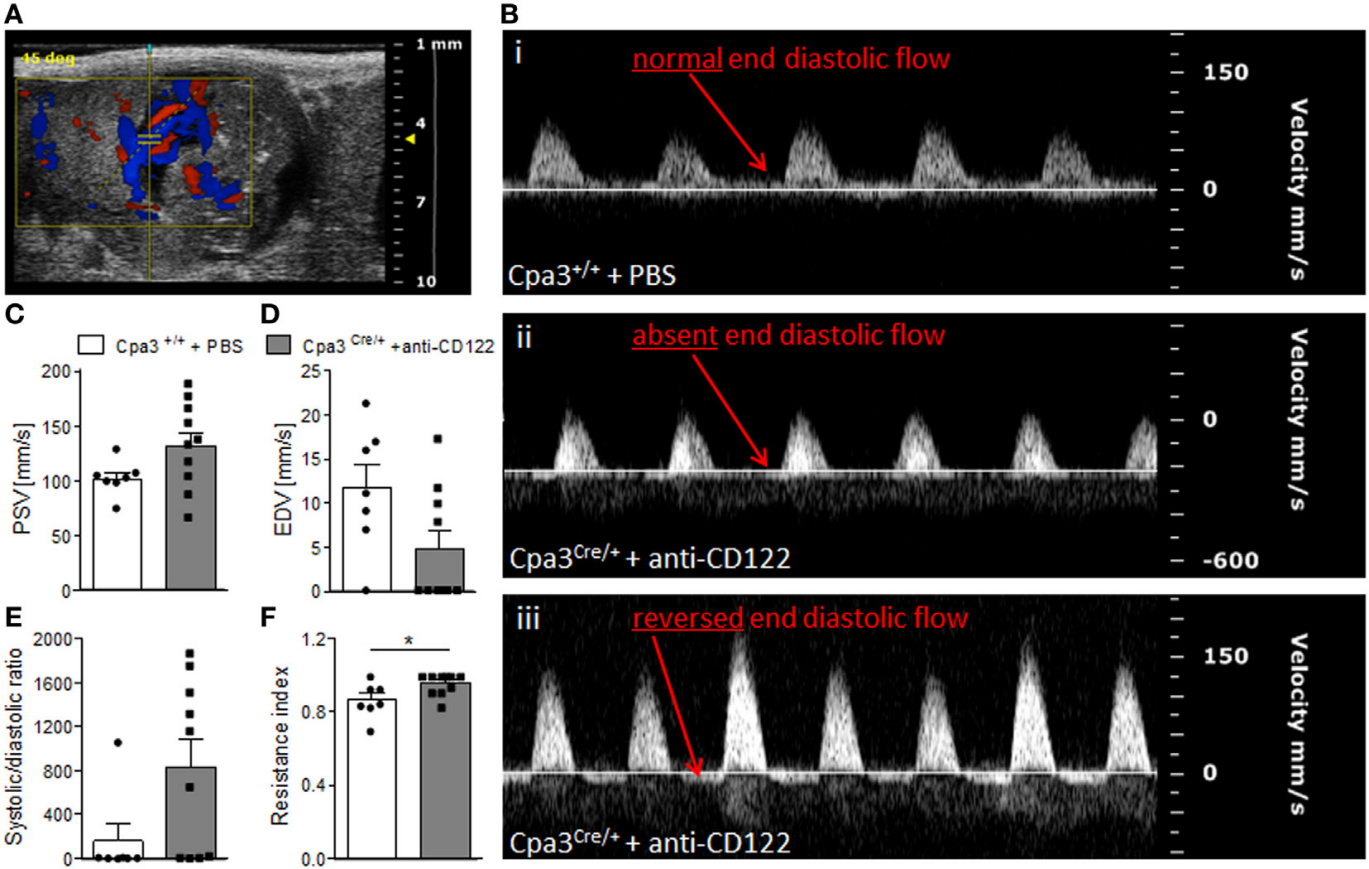
Analysis of umbilical artery velocities at gd14. **(A)** Representative Color Doppler image of a fetal UmA at gd 14. **(B)** Representative Pulse-wave Doppler images from Cpa3^+/+^ + PBS (i) and Cpa3^Cre/+^ + anti-CD122 (ii, iii) mice, showing normal end diastolic flow (i), absent end diastolic flow (ii), or reversed end diastolic flow (iii). PSV **(C)**, EDV **(D)**, systolic/diastolic ratio **(E)**, and resistance index **(F)** of UmAs of fetuses from Cpa3^+/+^ + PBS (mice *n* = 3, UmA measurements *n* = 7) and Cpa3^Cre/+^ + anti-CD122 (mice *n* = 3, UmA measurements *n* = 10) mice at gd14. Data are presented as mean with SEM. Statistical analysis was performed using the unpaired *t*-test (*P < 0.05). UmA, umbilical artery; gd, gestation day; PSV, peak systolic velocity; EDV, end diastolic velocity.

Next, we recorded UmA (Figure 5A) velocities at gd14 in fetuses from NK/MC-deficient vs control mice. Thereby, we measured the PSVs and EDVs to calculate the systolic/diastolic ratio and resistance index. This is relevant as increased sytolic/diastolic ratio, high resistence index, as well as a reduction or a missing detection of diastolic flow velocity as well as a reversed end diastolic flow in the UmA are all signs for IUGR (6, 20). WT mice always showed a normal end diastolic flow in UmAs (Figure 5B, i). In contrast, some fetuses from NK/MC-deficient mothers presented UmAs with absent end diastolic flow (Figure 5B, ii); in some cases even a reversed diastolic flow could be observed (Figure 5B, iii). Umbilical arteries of fetuses from NK/MC-deficient and WT control mice showed comparable PSVs (Figure 5C). In contrast, EDVs were strongly reduced in umbilical arteries of fetuses from NK/MC-deficient mice compared to WT (Figure 5D). This results in an increased systolic/diastolic ratio and a statistically significant increased resistance index of UmAs (*P* < 0.05) in fetuses from NK/MC-deficient mothers (Figure 5E) in comparison to WT.

## Discussion

Remodeling of SAs is a critical step in early pregnancy with serious consequences for fetal well-being and later life. During this step, thick-walled vessels transform into highly dilated, thin-walled vessels in order to adapt to the changing requirements during pregnancy. The mechanisms behind this remodeling can be divided into trophoblast-dependent and trophoblast-independent mechanisms (21). It is known that cells of the innate immune system, in particular uNKs and uMCs, can influence both mechanisms. uNKs positively influence SA remodeling by the secretion of mediators like vascular endothelial growth factor, placental growth factor, and interleukin-8 that positively influence trophoblast invasion. Interferon-γ regulates the expression of genes involved in cell adhesion, smooth muscle cell proliferation, and apoptosis as well as matrix metalloproteinase (MMP) expression. MMPs are in turn responsible for degradation of extracellular matrix compounds, an important step that occurs before trophoblast invasion. uMCs positively influence SA remodeling and placentation (17, 22, 23). If MCs are absent, SAs are not properly remodeled, placentas are too small or functionally impaired while the offspring is affected by IUGR (15, 17). Mcpt5, expressed by MCs, was found as a mediator able to induce the apoptosis of uterine smooth muscle cells, a key event during SA remodeling. *In vitro*, we could show that chymases foster trophoblast migration and invasion ability, another important mechanism during SA remodeling (17). Interestingly, the absence of one cell type in mouse models seems to be compensated by an augmentation in number of the other one (15), perhaps a physiological mechanism to ensure a proper SA remodeling and thus, adequate supply of the fetus with nutrients. Astoundingly, the simultaneous ablation of both NKs and MCs compromised the fetal growth and well-being of more than half of the progeny, pointing out the importance for the critical pregnancy step of SA remodeling. Having confirmed that the absence of NKs and MCs is directly related to IUGR, we next aimed to understand at which time point differences in size and development can be observed in uterus. Learning this is the first step to understand at which time point pregnancy is jeopardized in the absence of these cells and to design therapeutic strategies in pregnancy models aimed to normalize fetal growth. Here, we observed that implantation sizes were comparable between NK/MC-deficient and NK/MC-sufficient mice at gd5 and 8. First signs of growth restrictions were apparent at gd10, as we recorded smaller implantation sizes in MC/NK-deficient mice compared to the controls. The difference in size worsened at gd12. At gd14, the measurement of whole implantations *via* ultrasound did not provide reliable results, as implantations were too large to fit in screen and be captured for later measurements. The analysis of single organs and structures was, however, possible. Weighting placentas and fetuses at day 14, the last day of our experiment, confirmed IUGR. Thus, fetal growth retardation is initiated at latest at gd10, as we could confirm measureable differences by ultrasound. At this time, SA remodeling was also impaired as analyzed histologically (17). Smaller implantations could be observed from this gestational day onward, suggesting that insufficient SA remodeling is the cause of fetal growth restriction. Not only defects in SA remodeling but also defective placental development can be causative of IUGR. It has been shown that abnormal placental weight or placental insufficiency are associated with adverse pregnancy outcomes (24) and health consequences in early childhood as well as in later life (25–27). NKs are key regulators of placental development (28, 29); MCs are, as a matter of fact, also directly associated with placenta development (22, 23, 30). Mice lacking both, NKs and MCs had significantly reduced placental area, thickness and diameter as analyzed at gd10 and 12 compared to animals that have normal numbers of both innate immune cells. The FPI, a marker of placental insufficiency, was significantly higher in NK/MC-deficient mice compared to WT mice at all stages analyzed.

The embryonic UmA, that transports deoxygenated fetal blood from the fetus to the placenta, is a reference for feto–placental flow conditions. It is safe to state that the assessment of fetal well-being relies on the qualitative and quantitative assessment of UmAs. Here, dynamics of UmAs were analyzed to investigate whether the simultaneous ablation of uNKs and uMCs and impaired SA remodeling and placentation were associated with abnormal fetal blood vessel parameters. UmAs were evaluated by measuring PSVs and EDVs. Calculation of the systolic/diastolic ratio and resistance indexes followed. A reduced perfusion of the intravillous space of the placenta that can be caused by insufficient SA remodeling can lead to inadequate supply of the fetus. An inadequate supply is often compensated by centralization of the blood flow by the fetus, so that important organs like heart and brain get enough oxygen to develop correctly (6). A deterioration of the fetal blood circulation impacts first in a high systolic/diastolic ratio, it leads then to a reduced or absent diastolic flow and ends in a reversed end diastolic flow of the UmA (6). Interestingly, we observed these events in some fetuses from MC/NK-deficient mothers, namely lower EDVs, higher systolic/diastolic ratios and statistically significant higher resistance indexes compared to fetuses from WT mothers. The abnormal Doppler measurements included absent or reversed end diastolic flow.

As pregnancy advances and the placenta maturates, placenta vascular resistance decreases (8). Even though there are differences between the human and mouse placenta histology, many parameters, including placental perfusion dynamics are similar and comparable; thus studies in mice are particularly useful for elucidating putative mechanisms that can be altered in pregnancy pathologies. Abnormal uterine artery Doppler values have been shown to be associated with perinatal complications (31). For example, in women who develop preeclampsia, a pathological increase in placental vascular resistance that is previously detectable by abnormal Doppler can be documented (31). Similarly, Olofsson and colleagues reported defective SA remodeling in pregnancies complicated by fetal growth retardation and this was associated with increased UA flow resistance (32). Our NK/MC-deficient mice were affected by defective SA remodeling and IUGR, albeit being normotensive (17). Here, the evaluation of the UA was undertaken by registering PSV and EDV. Resistance indexes were calculated automatically by the Vevo 2100 System software. We found no differences between controls and MC/NK-deficient mice at gd10. Further investigations of UA parameters at different gestational days would be useful.

The strength of our study was the use of a technology that allows the measurement of *in vivo* parameters during pregnancy and the follow-up of single mice at relevant gestational days (gd5, 8, 10, 12, and 14). By using high frequency ultrasound, we could show that adverse fetal blood circulation and retardation of fetal growth at gestation day 10 are the events preceding the IUGR phenotype observed at birth. The knowledge of the mechanisms leading to abnormal fetal development and the underlying cause of IUGR is a milestone for the development of strategies to better monitor mothers that are at risk and to delineate treatment options to prevent the growth restriction.

## Ethics Statement

This study was carried out in accordance with the recommendations of the Ministery of Saxony-Anhalt, Germany. The protocol was approved by the “Landesverwaltungsamt Sachsen Anhalt: 42502-2-1296UniMD.”

## Author Contributions

Experiments were performed and analyzed by NM. NM substantially contributed to manuscript preparation. TS analyzed and discussed the data and contribute to manuscript preparation. AZ conceived the studies, supervised the work, wrote the paper, and provided financial support. All authors revised the manuscript and were involved in its final approval.

## Conflict of Interest Statement

The authors declare that the research was conducted in the absence of any commercial or financial relationships that could be construed as a potential conflict of interest.
